
JOURNAL INFORMATION
==============================
NLM Title Abbreviation: ScientificWorldJournal
Journal ID: ScientificWorldJournal
NLM Journal ID: 101131163
Journal ID: TSWJ

The Scientific World Journal

ISSN: 2356-6140
EISSN: 1537-744X
Publisher: Wiley

ARTICLE INFORMATION
==============================
PMCID: PMC5936594
PMID: 15674452
DOI: 10.1100/tsw.2005.15
Article ID: 981462
Article version: 1
Subjects: Commentary

TSUNAMI: Disconnect and the Aftermath

Manning William J.
University of Massachusetts, Amherst, USA
Publication date: 2005
Electronic publication date: 2005 Jan 23
Volume: 5
First page: 62
Last page: 63
Received 2005 Jan 14; Accepted 2005 Feb 14
Copyright: Copyright © 2005 William Manning.
Copyright year: 2005
License: This is an open access article distributed under the Creative Commons Attribution License, which permits unrestricted use, distribution, and reproduction in any medium, provided the original work is properly cited.
License URL: https://creativecommons.org/licenses/by/3.0/

Keywords: oceans physics, terrestrial systems, marine systems

==============================
Commentary
*TheScientificWorldJOURNAL* (2005) 5, 62–63
ISSN 1537-744X; DOI 10.1100/tsw.2005.15

### TSUNAMI: Disconnect and the Aftermath

William J. Manning
*University of Massachusetts, Amherst*

**E-mail:** wmanning@microbio.umass.edu

*Received January 14, 2005; Accepted January 14, 2005; Published January 23, 2005*

**KEYWORDS:** earthquakes, floods, tsunamis, tourism, agriculture, fishing

**DOMAINS**: oceans physics, terrestrial systems, marine systems

At one time, few people lived permanently near the edges of the world's oceans. Now, more than half of the world's population lives near beaches and estuaries. People in developed countries have primary or secondary homes there to enjoy the views and for recreation. Poor people in developing countries live there to engage in fishing, jobs in tourism for people from developed countries, and for subsistence farming in river delta areas. They remain despite persistent hurricanes, typhoons, and resulting flooding. There is a serious disconnect for both rich and poor people between the reality of living near oceans and the danger that this entails. For a variety of reasons, people are becoming less and less connected to the world around them. Natural events that have devastating effects on humans are considered to be strange and unusual, rather than a normal part of human existence on a continually changing planet. Our disconnect from nature has serious consequences.

Earthquakes under the oceans, from shifting tectonic plates or volcanic eruptions, are known to result in oceanic disturbances that result in elevated wave activity near ocean shores. A high wall of water, or wave, that emerges from the water column caused by an ocean disturbance is called a tsunami (Japanese for "harbor wave"). Globally, 80% of tsunamis occur in the Pacific Ocean region. Seismic waves above 7.5 m occur there naturally every 15 years. Tsunamis are not unusual events. When I was in Japan recently, I discovered that they are a favorite theme in Japanese art, especially in woodblock prints.

The earthquake that recently occurred in the Indian Ocean was unusually strong (9.0 on the Richter Scale) and the resulting tsunami resulted in horrific loss of life and property in countries like Indonesia, India, and Sri Lanka. Television has enabled everyone to witness the devastation and its aftermath. More than 150,000 people have died and huge coastal areas have been destroyed. Stunned local populations are responding to an enormous outpouring of international relief aid.

What will be the aftermath of this devastating tsunami? Reefs have been damaged or destroyed as have coastal mangrove areas and estuaries. What effects this will have on fish populations and reproduction are unknown. Ponds used to cultivate shrimp have been destroyed. Few fishing boats are left to harvest what fish may be left. Seawater was driven inland, killing plants and animals, and infiltrating agricultural land and wells used for drinking water. The long-term effects on agriculture and well water are not known.

Ecologists have an opportunity to study recolonization of affected areas by plants and animals. Epidemics of diseases have not developed as prompt burial of bodies and distribution of clean water and

©2005 with author.

food seem to have averted this danger. Rainy weather may result in abundant mosquito populations and the threat of malaria. This will prompt widespread spraying of insecticides, like DDT. While necessary, this will contribute to the plume of insecticides that already flows out of Asia to the South Pacific region. Tourism will probably be re-established quickly, but the future of previously populated areas, agriculture, and fishing remains open to question.

Despite loss of life and physical and social disruption, it is possible to consider that earthquakes and volcanic eruptions and resulting tsunamis are inevitable recurring events that may have some long-term ecological benefits. Seismic waves can lift nutrient-rich estuarine and river delta sediments and redistribute them across coastal agricultural areas and result in increased soil fertility. Volcanic eruptions release nutrients to surrounding areas and add to the level of carbon dioxide in the troposphere, which ensures an environment warm enough to enable plants, animals, and humans to exist and plants to engage in photosynthesis.

### REFERENCES

1. Anon. (2005) After the deluge. *Economist* **374(8048),** 23–25.
2. Broad, W.J. (2005) “Deadly and Yet Necessary Quakes Renew the Planet.” *New York Times* January 11. pp. D1, D4.
3. Dean, C. (2005) “A Modern Peril: Living Near the Jaws of the Sea. *New York Times* January 11. p. D4.
4. Hopkin, M. (2005) Triple slip of tectonic plates caused seafloor surge. *Nature* **433**, 3.
5. Quirin, S. (2005) Tsunamis: a long-term threat. *Nature* **433,** 4.

**This article should be referenced as follows:**

Manning, W. (2005) TSUNAMI: Disconnect and the aftermath. *TheScientificWorldJOURNAL* **5**, 62–63.
